# Saline Injections in Infantile Diarrhœa

**Published:** 1913-07-26

**Authors:** J. Roberson Day

**Affiliations:** 31 Devonshire Place, Wimpole Street, W.


### THE EDITOR'S LETTER-BOX.

### Saline Injections in Infantile Diarrhœa.

*To the Editor of* The Hospital.

Sir,—My attention has been called to your article on "Saline Injections in Infantile Diarrhœa" in to-day's issue. If you refer to my letters which appeared, at the time of the severe epidemic of gastro-enteritis, in the *British Medical Journal*, you will see that the sea-water injections were most remarkably successful in these cases, and in some instances succeeded after the normal saline fluid (Ringer's) had been used without success.

The tone of your article is unfortunate. We physicians are all trying to do our best to cure disease, and in this particular case a small party of medical friends, "the small knot" you refer to, stimulated by an article in the *Medical Annual,* set out for Paris to see the work of Quinton as carried on at the two large "Dispensaires Marines." I can only say what we saw done there made us wish to give the same benefits to our poor children in London. We returned and were successful in opening the Quinton Institute in Poland Street, Soho. Almost immediately we had to deal with the epidemic of two years ago, and the results of the treatment brought us patients from far and wide. The work is still going on, and qualified practitioners who are interested are always welcome. The medical staff are honorary. You say "unsatisfactory as several aspects of this incident were." May I ask what you mean by this? We all know the press got hold of the treatment and seized the opportunity for "copy" when there was little of interest in the papers.

Again quoting your article: "The hypothesis upon which the much-vaunted Quinton treatment is based has no rational basis whatever, and is palpably absurd." Pardon me, you are mistaken, and I refer you to the scientific work of M. René Quinton, with which you are evidently unacquainted.

The *cost* of the Plasma de Quinton, I agree, is the great bar to its use, and we are seeking and now using a modified sea-water, identical in composition, prepared by an English firm, and obtained from English seas. I repeat my invitation to all who are interested—Come and see; and to you, Sir, in particular before you next attempt to criticise—Yours faithfully,

J. Roberson Day, M.D. (Lond.), etc.

31 Devonshire Place, Wimpole Street, W.
July 19, 1913.

[Dr. Day is mistaken in thinking that we are unacquainted with the nature of M. Quinton's hypothesis, which we still believe to deserve the adjectives we applied to it. The article in the *Medical Annual* to which he refers is perfectly familiar to us. In our turn we would refer him to the article by his namesake, Professor Day of Cairo, in a recent number of the *Practitioner*. Our comments were based on the results disclosed in that article, which Dr. J. R. Day discreetly ignores. Dr. Day wants to know what we mean by a statement we made. If he will refer to The Hospital, September 9, 1911, p. 587, and September 30, 1911, p. 667, he will find our meaning fully explained.—Ed. The Hospital.]

